# A novel benzofuran derivative, ACDB, induces apoptosis of human chondrosarcoma cells through mitochondrial dysfunction and endoplasmic reticulum stress

**DOI:** 10.18632/oncotarget.13171

**Published:** 2016-11-07

**Authors:** Chen-Ming Su, Chien-Yu Chen, Tingting Lu, Yi Sun, Weimin Li, Yuan-Li Huang, Chun-Hao Tsai, Chih-Shiang Chang, Chih-Hsin Tang

**Affiliations:** ^1^ Department of Biomedical Sciences Laboratory, Affiliated Dongyang Hospital of Wenzhou Medical University, Dongyang, Zhejiang, China; ^2^ Graduate Institute of Basic Medical Science, China Medical University, Taichung Taiwan; ^3^ Graduate Institute of Pharmaceutical Chemistry, China Medical University, Taichung, Taiwan; ^4^ Department of Cardiology, Affiliated Dongyang Hospital of Wenzhou Medical University, Dongyang, Zhejiang, China; ^5^ Department of Biotechnology, College of Health Science, Asia University, Taichung, Taiwan; ^6^ School of Medicine, China Medical University, Taichung, Taiwan; ^7^ Department of Orthopedic Surgery, China Medical University Hospital, Taichung, Taiwan; ^8^ Department of Pharmacology, School of Medicine, China Medical University, Taichung, Taiwan

**Keywords:** benzofuran, chondrosarcoma, apoptosis, endoplasmic reticulum (ER) stress, mitochondrial dysfunction

## Abstract

Chondrosarcoma is one of the bone tumor with high mortality in respond to poor radiation and chemotherapy treatment. Here, we analyze the antitumor activity of a novel benzofuran derivative, 2-amino-3-(2-chlorophenyl)-6-(4-dimethylaminophenyl)benzofuran-4-yl acetate (ACDB), in human chondrosarcoma cells. ACDB increased the cell apoptosis of human chondrosarcomas without harm in chondrocytes. ACDB also enhanced endoplasmic reticulum (ER) stress, which was characterized by varieties in the cytosolic calcium levels and induced the expression of glucose-regulated protein (GRP) and calpain. Furthermore, the ACDB-induced chondrosarcoma apoptosis was associated with the upregulation of the B cell lymphoma-2 (Bcl-2) family members including pro- and anti-apoptotic proteins, downregulation of dysfunctional mitochondria that released cytochrome C, and subsequent activation of caspases-3. In addition, the ACDB-mediated cellular apoptosis was suppressed by transfecting cells with glucose-regulated protein (GRP) and calpain siRNA or treating cells with ER stress chelators and caspase inhibitors. Interestingly, animal experiments illustrated a reduction in the tumor volume following ACDB treatment. Together, these results suggest that ACDB may be a novel tumor suppressor of chondrosarcoma, and this study demonstrates that the novel antitumor agent, ACDB, induced apoptosis by mitochondrial dysfunction and ER stress in human chondrosarcoma cells *in vitro* and *in vivo*.

## INTRODUCTION

Of all the malignant bone tumors, chondrosarcoma occurs as an *in situ* secondary incidence [1]. Currently, the treatment of chondrosarcoma involves the use of chemotherapy or radiation therapy, but its management is a complicated challenge because of its unresponsive nature [2]. Clinically, chondrosarcoma possesses a poor prognosis which lack an effective adjuvant treatment so that surgical resection is the major therapy for this mesenchymal malignancy [3]. Therefore, exploring a novel and rare side-effect strategy might be critical for the treatment of chondrosarcoma.

Reactive oxygen species (ROS) are originated with the metabolism of oxygen exhaustion. Aerobic respiration produces adenosine triphosphate (ATP) and other harmful superoxide anion radical (O_2_^−^), which can then form other ROS such as highly reactive hydroxyl radicals and hydrogen peroxide (H_2_O_2_) [4, 5]. As excess ROS or antioxidant depletion leads to disruption of balance from aerobic respiration, oxidative stress would occur. Accumulating evidence demonstrates that chemotherapy may be selectively toxic to tumor cells owing to increasing stressed cells over limitation and oxidant stress [6, 7]. In addition, activation of the mitochondria-dependent apoptosis signaling triggered ROS signaling through the apoptotic signaling proteins, such as BH3 interacting-domain death agonist (Bid), B-cell lymphoma-extra large (Bcl-XL), B cell lymphoma-2 homologous antagonist/killer (Bak), B cell lymphoma-2 associated-X protein (Bax), or B cell lymphoma-2 (Bcl-2) with permeabilization and cell death of mitochondrial membrane [4, 8]. Nevertheless, involvement of ROS and mitochondrial dependent signalings in chondrosarcoma needs to be further clarified.

As central organelle, the endoplasmic reticulum (ER) is responsible for lipid synthesis and protein folding, modification, and maturation. Due to the broken ER function, ER stress derives from various toxic distractions including protein misfolding, hypoxia, and Ca^2+^ overload [9–11]. Accumulating evidence indicates that ER stress plays an important role in the apoptosis regulation and connected with calcium-dependent signaling pathways and unfolded protein response [12, 13]. In addition, glucose-regulated proteins (GRPs), the primary glycoproteins, play a critical role in the ER including GRP78 and GRP94 against oxidative injury and regulate ribozyme approaches [14–16]. The induction of GRPs for antiapoptotic function may cause drug resistance and cancer development [17, 18].

Benzofuran appears structurally like natural products and functions as human protein kinase inhibitors [19]. Recently, benzofuran has been reported the role of antiproliferative activity in tumors especially against p53-independent malignant tumors [20]. The roles of benzofuran derivative in chondrosarcoma remain largely unknown. Therefore, in this study we synthesized a brand-new benzofuran derivative, 2-amino-3-(2-chlorophenyl)-6-(4-dimethylaminophenyl)benzofuran-4-yl acetate (ACDB), and evaluated the antitumor role of ACDB in response to human chondrosarcoma cells. We attempt to investigate ACDB antitumor activity and explore the mechanism by which it induces chondrosarcoma apoptosis.

## RESULTS

### ACDB enhanced human chondrosarcoma cells apoptosis

**Figure 1:**
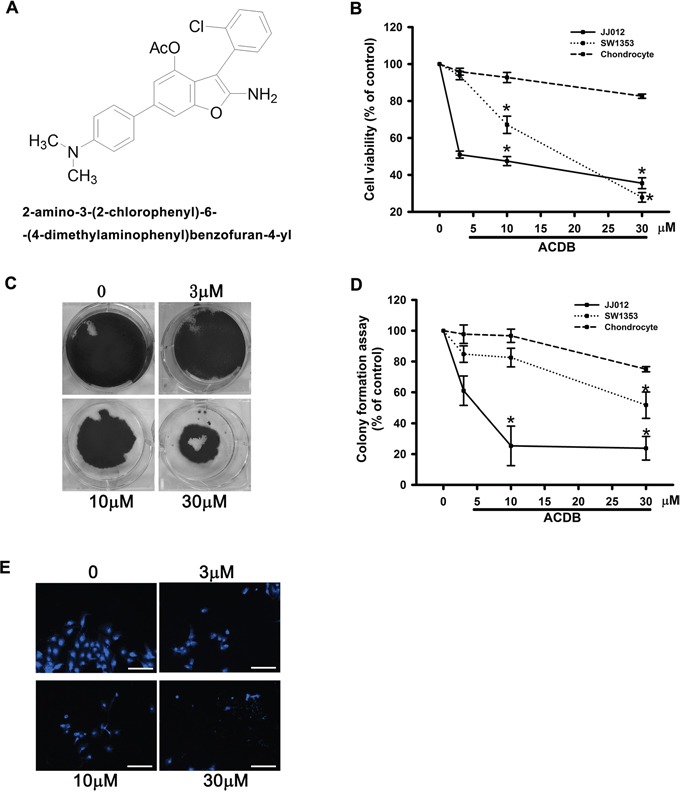
The effect of ACDB on cell viability and formation and colony assay in human chondrosarcoma cells **A.** Novel structure of benzofuran derivative, 2-amino-3-(2-chlorophenyl)-6-(4-dimethylaminophenyl)benzofuran-4-yl acetate; ACDB. **B.** Cells were incubated with various concentrations of ACDB for 48 h, and the cell viability was examined by MTT assay (n=6). **C.** For the colony-forming assay, the clonogenic assay was performed as described in Materials and Methods section. **D.** The quantitative data of colony-forming assay. **E.** JJ012 cells were incubated with ACDB for 48 h, and stained with DAPI (scale bar = 5 μm). Results are expressed as the means ± SEM of four independent experiments. * *P* < 0.05 as compared with control group.

For the cytotoxic investigation of ACDB, we first examined its effects on the survival between human chondrosarcoma cell lines and normal chondrocytes with the MTT assay. Both chondrosarcoma cell lines and normal chondrocytes were treated with ACDB (3, 10, 30 μM) that triggered cell apoptosis of JJ012 and SW1353 cell lines with half-maximal inhibitory concentration (IC50) values of 4.9 and 19.1 μM, respectively (Figure 1B). The role of ACDB in anticancer activities was further performed using clonogenic assays (Figure 1C), which is connected with prior *in vivo* tumorigenicity assays in nude mice [27]. While the JJ012 cells formed clones in the untreated control wells (Figure 1D), treatment with ACDB (3, 10, 30 μM) induced a dose-dependent inhibition of clonogenicity, and the quantitative results containing normal condrocytes are shown in Figure 1D. Interestingly, ACDB led to the cell viability of normal chondrocytes without impairs in response to MTT and clonogenic assays (Figure 1B and 1D, dashed line). Furthermore, JJ012 cells treated with ACDB significantly induced the condensation of chromatin as exhibited by the DAPI staining (Figure 1E).

**Figure 2:**
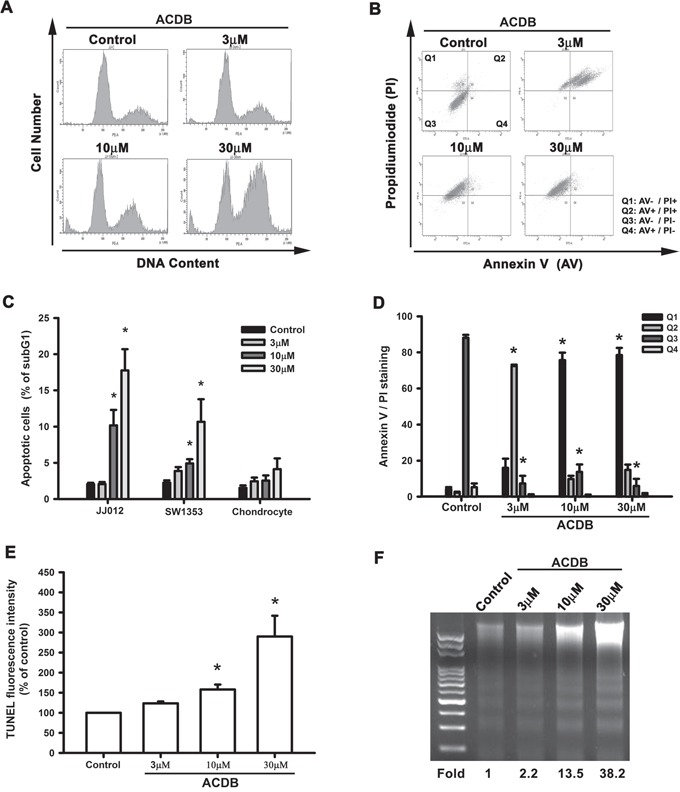
ACDB induces apoptosis in human chondrosarcoma cells **A** and **C.** Cells were treated with vehicle or ACDB for 48 h. The cell cycle analysis (PI staining) was examined and quantified by flow cytometry. **B** and **D.** JJ012 cells were treated with vehicle or ACDB for 48 h. The percentage of apoptotic cells was analyzed by flow cytometry of Annexin V/PI double staining, and the results were quantified. **E.** Cells were treated with vehicle or ACDB for 48 h. The TUNEL positive cells were examined by flow cytometry (n = 4). **F.** For measuring apoptosis, the DNA fragmentation assay was performed as described in Materials and Methods section. Results are expressed as the means ± SEM of four independent experiments. * *P* < 0.05 as compared with control group.

Next, we investigated if ACDB resulted in cell apoptosis of chondrosarcoma cells via various apoptotic assays. Treating cells with ACDB enhanced a dose-dependent production of cell death, causing sub G1 phase ascent of the cell cycle (Figure 2A). Thus, ACDB failed to affect sub G1 phase on normal chondrocytes (Supplementary Figure S1). To detect the cell death by using double-stained Annexin V/PI, a high proportion of Annexin V+ labeling was detected in the ACDB-treated cells compared to the control group (Figure 2B); quantitative data of the sub G1 phase and Annexin V/PI assay are shown in the Figure 2C and 2D, suggesting that ACDB probably induced late cell death during programmed cell death. In addition, those stimulated with ACDB exhibited a high increase TUNEL fluorescent intensity compared to JJ012 cells without treatment (Figure 2E). Then, we examined the DNA fragmentation, which provides information about the type of cell death and the pathways activated in the dying cells, and is a hallmark of apoptosis in biochemistry [21, 22]. As shown in Figure 2F, ACDB promoted DNA fragmentation in a dose-dependent increase (~2.2 to 38 fold) in JJ012 cells. Above results demonstrate that ACDB largely enhanced chondrosarcoma cell apoptosis through various different levels.

### Both reactive oxygen species and mitochondrial dysfunction contribute to cell apoptosis of ACDB in chondrosarcoma cells

**Figure 3:**
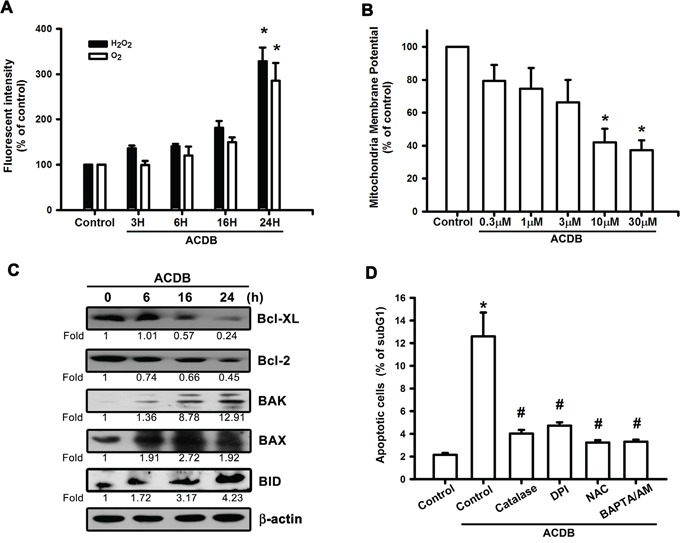
ACDB induces ROS production and mitochondrial dysfunction in human chondrosarcoma cells **A.** JJ012 cells were incubated with ACDB for different time intervals, the production of O2^−^ and H_2_O_2_ were examined by flow cytometry. **B.** JJ012 cells were incubated with various concentration of ACDB for 48 h, the mitochondrial membrane potential was examined by flow cytometry. **C.** JJ012 cells were incubated with ACDB for different time intervals, the Bax, Bak, BID, Bcl-2 and Bcl-XL expression were examined by Western blot analysis. **D.** JJ012 cells were pre-treated for 30 min with catalase (10,000 U/ml), NAC (4 μM), DPI (10 μM) or BAPTA-AM (10 μM) followed by stimulation with ACDB for 48 h, the percentage of apoptotic cells was analyzed by flow cytometry. Results are expressed as the means ± SEM of four independent experiments. *P < 0.05 as compared with control group; ^#^*P* < 0.05 compared with the ACDB-treated group.

ROS generation has been reported to play a crucial role of various anticancer agents in the proapoptotic activities [23, 24]. Therefore, we continued to examine whether ROS accumulation is involved in the ACDB-increased cell death. After treatment with ACDB, the JJ012 cells showed increased intracellular O_2_^−^ and H_2_O_2_ levels, detected using the DHE- and H_2_DCFDA-based FACS assays, respectively (Figure 3A). Next, we further continued to explore the mitochondrial dysfunction by using a specific membrane potential dye. As shown in Figure 3B, mitochondria membrane potential was significantly decreased in a concentration-dependent trend after ACDB exposure to chondrosarcoma cells. The expression of the mitochondrial membrane-dependent family proteins was measured. Once mitochondrial dysfunction occurred, ACDB increased protein levels of Bak (12.9 fold), Bid (4.23 fold) and Bax (1.92 fold) and decrease Bcl-XL (0.24 fold) and Bcl-2 (0.45 fold) protein expression in response to chondrosarcoma cell apoptosis (Figure 3C). In numerous abnormal situations, the production of ROS derived from a critical enzyme, nicotinamide adenine dinucleotide phosphate (NADPH) oxidase [25]. Pretreatment of cells with DPI, catalase, or NAC abrogated the ACDB-induced ROS production and cell apoptosis (Figure 3D), suggesting that ACDB-induced cell apoptosis involves ROS release in chondrosarcoma. Above results indicate that ACDB enhanced chondrosarcoma cells apoptosis via ROS and mitochondrial dysfunction.

### ACDB caused calcium accumulation and ER stress

**Figure 4:**
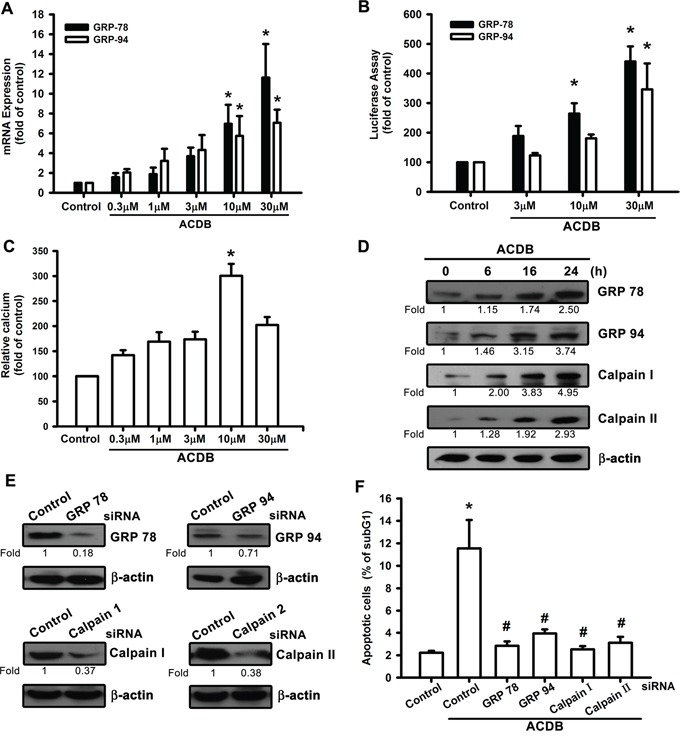
ER stress-related proteins activation are involved in ACDB-mediated cell apoptosis in human chondrosarcoma cells **A** and **B.** JJ012 cells were incubated with ACDB, and mRNA expression and GRP promoter were examined by qPCR and luciferase assay, respectively. **C.** JJ012 cells were incubated with ACDB for different concentration. The Ca^2+^ flux was examined by flow cytometry. **D.** JJ012 cells were incubated with ACDB for different time intervals, the GRP78, GRP94, calpain I and calpain II expression were examined by Western blot analysis. **E.** Cells were transfected with siRNA against GRP78, GRP94, calpain I, calpain II or control for 24 h before incubated with ACDB. GRP78, GRP94, calpain I and calpain II expression was examined by Western blot analysis. **F.** Cells were transfected with siRNA against GRP78, GRP94, calpain I, calpain II, or control for 24 h and then stimulated with ACDB for 48 h, and the percentage of apoptotic cells was analyzed by flow cytometry of PI-stained cells. Results are expressed as the means ± SEM of four independent experiments. *P < 0.05 as compared with control group; ^#^*P* < 0.05 compared with the ACDB-treated group.

A previous report demonstrated that mitochondrial dysfunction may lead to pancreatic β-cell apoptosis through ER stress and GRP regulation [26]. For the involvement of GRP in chondrosarcoma cells apoptosis, the levels of the GRP78 and GRP94 were investigated that ACDB induced their mRNA expression (Figure 4A) and promoter-luciferase activity (Figure 4B). The accumulation of ER stress originated from the depletion of luminal ER calcium stores [27], and Ca^2+^ mobilization was investigated in response to ACDB-induced cell apoptosis. After treated with ACDB in JJ012 cells, the Ca^2+^ level was significantly increased compared to control group (Figure 4C), and the BAPTA-AM, the Ca^2+^ chelator, decreased the ACDB-induced cell apoptosis (Figure 3D), suggesting that Ca^2+^ production is involved in the ACDB-promoted chondrosarcoma cell apoptosis.

In addition, calpains are critical enzymes in the intracellular signaling cascades and potential mediators of calcium-induced apoptosis [28]. ACDB markedly increased protein levels of GRPs and calpains in a time-dependent manner (~2.5 to 4.95 fold) (Figure 4D). To further substantiate the functional roles of GRPs and calpains in ACDB-induced apoptotic cell death, we examined its effects on siRNA against GRP-78, GRP-94, calpain I, and calpain II. Transfection of chondrosarcoma cells for 12-16 h with these siRNAs not only reduced the protein expression (~0.71 to 0.18 fold) (Figure 4E), but it also antagonized the ACDB-induced cell apoptosis (Figure 4F). Therefore, above results indicate that ACDB-induced apoptosis results in ER stress including GRP and calpain activation through mitochondrial dysfunction.

### ACDB increased caspase-dependent signaling in chondrosarcoma cells

**Figure 5:**
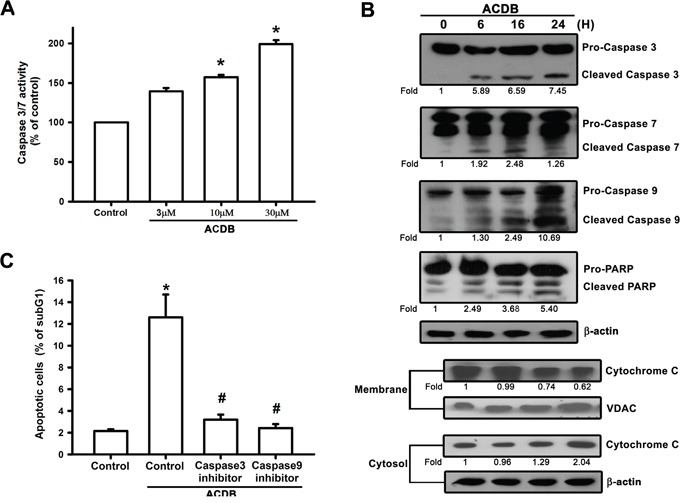
ACDB induces the activation of caspase in human chondrosarcoma cells **A.** JJ012 cells were incubated with ACDB for 24 h. Caspase-3/7 activities were examined by caspase activity kit. **B.** JJ012 cells were incubated with ACDB for different time intervals. Levels of PARP, cytochrome C, caspase-3, caspase-7 and caspase-9 expressions were examined by Western blot analysis. Results are expressed as the means ± SEM of four independent experiments. **C.** Cells were pretreated for 30 min with z-DEVD-FMK (caspase 3 inhibitor) or z-LEHD-FMK (caspase 9 inhibitor), followed by stimulation with ACDB for 24 h. The percentage of apoptotic cells was the analyzed by flow cytometry of PI-stained cells. *P < 0.05 as compared with control group; ^#^*P* < 0.05 compared with the ACDB-treated group.

A previous apoptosis-associated study reported that caspase activation derived from ubiquitous calpains within ER stress [29]. We continued to clarify the apoptotic function of ACDB in chondrosarcoma that caspase-3/7 activity and protein expression (~1.26 to 7.45 fold) were elevated after ACDB exposure in JJ012 cells (Figure 5A and 5B, upper panel). Furthermore, treatment with ACDB increased the expression of the upstream protein caspase-9 (10.89 fold) and the cytosolic fraction of cytochrome C (2.04 fold) in JJ012 cells (Figure 5B) as well as cleaved-PARP expression (5.40 fold) (Figure 5B). On the other hand, we continued to determine the involvement of caspase with the specific caspase-3 inhibitor, z-DEVD-FMK, and caspase-9 inhibitor, z-LEHD-FMK [30]. JJ012 cells pretreated with z-DEVD-FMK and z-LEHD-FMK largely diminished ACDB-enhanced cell apoptosis, as shown by the cell cycle assay (Figure 5C). Therefore, our data suggest that caspase-3/7, caspase-9, and cleaved-PARP activation is involved in the ACDB-mediated human chondrosarcoma cell apoptosis.

### ACDB retarded tumor growth in a murine xenograft model

According to the ACDB-enhanced apoptotic effect *in vitro*, we continued to exhibit the antitumor activities of ACDB *in vivo*. We performed mouse xenograft model *in vivo* followed by treatment with ACDB for 20 days. Mice were sacrificed at the end of the treatment and the tumors were collected.

**Figure 6:**
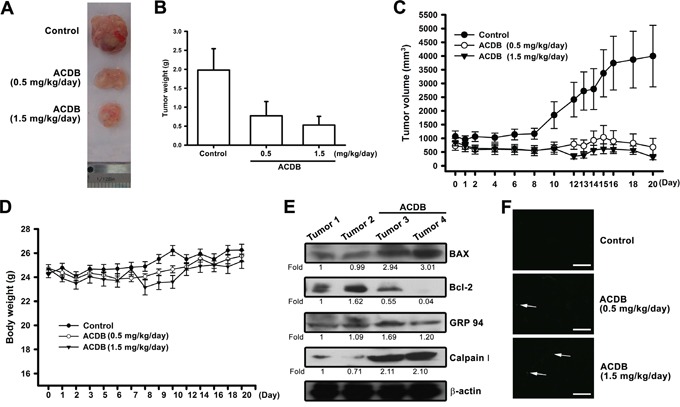
ACDB inhibits tumor growth in nude mice **A** and **B.** Mice were injected subcutaneously with JJ012 tumor cells. After the tumors reached 1000 mm^3^ in size, ACDB (0.5 or 1.5 mg/kg) or vehicle was administered daily for 3 weeks. **C** and **D.** Mean tumor volume and body weight were measured at the indicated number of days after implantation (n=10). **E.** Western blot analysis determined protein levels of BAX, Bcl-2, GRP94, and calpain I from the tumors with and without ACDB treatment. **F.** TUNEL assay in tissues from chondrosarcoma cells xenografts. Apoptosis happened only in approximately 1% of tumor tissue in the control group, while ACDB-treated tumors show marked green staining of fragmented nuclei, indicative of apoptosis (scale bar = 50 μm).

ACDB significantly diminished xenograft tumor size and weights (Figure 6A and 6B) and tumor volume and body weights were estimated once two days (Figure 6C and 6D) compared to the control group, indicating that ACDB produced a dose-dependent inhibition of tumor growth without toxicity. Furthermore, the *ex vivo* tumor sections analysis showed that the protein levels of Bax, Bcl-2, GRP-94, and calpain I increased (~1.2 to 3.01 fold) or decreased (~0.55 to 0.04 fold) significantly in the ACDB-stimulated group than the control group (Figure 6E). In addition, an increase in TUNEL+ cells was observed in tumors of the ACDB-treated mice compared with that of tumors from the vehicle-treated mice (Figure 6F). Above results indicated that ACDB treatment strongly suppressed JJ012 xenograft tumor growth *in vivo* through regulation Bax, Bcl-2, GRP-94, and calpain I signaling (Supplementary Figure S2).

## DISCUSSION

In contrast to other mesenchymal malignancies such as osteosarcoma and Ewing's sarcoma that have demonstrated a thrilling increase in the long-term survival of patients with the approach of effective chemotherapy, chondrosarcoma continues to possess a poor prognosis due to lack of promising adjuvant therapies [31]. Therefore, to explore a novel therapeutic agent targeting the malignant survival of chondrosarcoma would be crucial for ameliorating the prognosis of patients. Benzofuran derivative has recently been reported to show anticancer activity in breast and cervical cancer [32, 33]. However, the antitumor activity of benzofuran derivative in chondrosarcoma cells are mostly unknown.

Therefore, in this study, we synthesized a new 2-amino-3-(2-chlorophenyl)-6-(4-dimethylaminophenyl)benzofuran-4-yl acetate, ACDB as an attractive lead compound with potential anticancer activity against human chondrosarcoma cells and good pharmacological properties. We first discovered that this benzofuran derivative induced a significant cell death in JJ012 than SW1353 cell line. Although both JJ012 and SW1353 are both grade II chondrosarcoma cells, however, the sensitivity from ACDB treatment in these two cell lines requires to be further discussed in the future. The chondrocytes appeared to have a greater resistance to apoptosis-inducing compounds than the other cell lines, and ACDB showed greater anticancer potential against human chondrosarcoma cells. Moreover, the metabolic rates of the primary chondrocytes and chondrosarcoma are different, which may explain their differential sensitivities to ACDB treatment.

It has been reported that several anticancer agents induced an enhancement of oxidative stress and connected with the apoptotic effects [34, 35]. ROS leads to cell apoptosis through various mechanism including initiation of mitochondrial permeability transition pore opening, the release of pro-apoptotic factors, and activation of caspase cascade [36]. The O_2_^−^ radical has also been shown to regulate Bcl-2-related protein expression, and the inhibition of O_2_^−^ by ρ-benzoquinone, the O_2_^−^ scavenger, prevents tumor apoptosis [37]. In this study, we found that ACDB triggered oxidative stress via activation of the O_2_^−^ and H_2_O_2_ production, and the role of NADPH oxidase was further investigated in ACDB-induced cell apoptosis. We used catalase (H_2_O_2_ scavenging enzyme), NAC (a direct scavenger of ROS), and DPI (an NADPH oxidase inhibitor) to determine ACDB-increased cell apoptosis, indicating that ROS accumulation contributes to ACDB-induced cell death of human chondrosarcoma.

The mitochondria-dependent apoptosis has been described as a pivot in the downstream signaling cascade of ROS during apoptotic process [38–40]. As our results of mitochondrial dysfunction did not occur until the cell was treated with ACDB for 48 h. Thus, the relationship between ROS and mitochondrial dysfunction needs to be further analysis in the future. Some important events have been noted in apoptosis involving mitochondrial dysfunction [41]. Cell apoptosis triggered various function of Bcl-2 family members that reveals the permeabilization of mitochondrial outer-membrane and relieves subsequently cytochrome C into the mitochondrial intermembrane space and cytosol [42]. In this study, we demonstrated that ACDB reduced the mitochondrial membrane potential and increased the release of cytochrome C. Nevertheless, the balance between anti- and pro-apoptotic proteins, which regulates the mitochondria-dependent apoptosis mediated by the Bcl-2 family members modulates cell life-and-death decisions [43]. We discovered that ACDB treatment significantly increased Bax, Bak, and Bid expression while it decreased that of Bcl-2 and Bcl-XL, suggesting that variations in the ratio of pro- and anti-apoptotic Bcl-2 family proteins might contribute to the apoptotic activity we observed. In agreement with these observations, we noted that the mitochondrial dysfunction might be engaged in the ACDB-increased apoptosis of human chondrosarcoma cells.

Calcium has been reported to play an important role in activating apoptosis [44], and release of calcium stores from the ER can activate caspase and contribute to apoptosis [45]. Under a variety of stressful conditions, the accumulation of unfolded or misfolded proteins in the ER leads to the onset of ER stress [46]. Our study found that ACDB-mediated cell apoptosis not only enhances cytosolic calcium levels but also i by the chelator, BAPTA-AM, in human chondrosarcoma cells. This suggests that ACDB induced apoptotic cell apoptosis via the activation of ER stress in human chondrosarcoma cells.

The upregulation of GRP78, which is another major indicator of ER stress, is believed to increase the buffering capacity of the body against stressful insults initiated by the ER [47]. Moreover, the roles of GRP94 in protein folding, calcium binding, targeting to endoplasmic-reticulum-associated protein degradation, and protective functions have been understood and observed in resistance to radiation in cervical cancer [48, 49]. Notably, we demonstrated in this study that ACDB increased GRP protein and mRNA expression and promoter activity, which was revealed by the GRP siRNA-induced antagonism of ACDB-mediated cell apoptosis potentiation. Similar functions have been reported with the observation that mitochondrial dysfunction induces ER stress and GRP94 protein expression in apoptosis of pancreatic β-cells [26, 50], and curcumin induces apoptosis of human lung carcinoma cells through GRP78 upregulation [51]. Therefore, these results suggest that ACDB increased GRP94 expression through mitochondrial dysfunction, and GRP78 transcription activity, which may play a pro-apoptotic role in the observed apoptosis.

Two kinds of cysteine proteases containing calpains and caspases regulate pathological cell apoptosis [52]. Although both caspase and calpain share their death-related substrates to regulate cells apoptosis [51], the effects of the ACDB on these two proteases families remain unclear. Our results indicate that calpains, caspase-3/7, and caspase-9 activation is involved in the ACDB-mediated human chondrosarcoma cell apoptosis. Collectively, above findings indicate that ACDB induced apoptotic cell death through ER stress and caspase-dependent pathways in human chondrosarcoma cells.

In conclusion, our findings demonstrate that the novel benzofuran derivative, ACDB, triggered the apoptosis of human chondrosarcoma cells *in vitro* and *in vivo*. Furthermore, ACDB-mediated chondrosarcoma apoptosis through the ROS release led to mitochondrial dysfunction and likely included caspase-regulated mechanisms. ACDB also regulated ER stress, GPR78 activation, and Ca^2+^ release, which subsequently triggered calpains, caspase cascade, ultimately leading to apoptosis. Although we have previously published a similar pathway in the treatment of chondrosarcoma using another compound, trichodermin [41], the effects of these two on chondrosarcoma need to be further analyzed in the future. Our proposed mechanisms of this antitumor activity remain to be further investigated, and the molecular basis of these observed effects may provide help during the development of novel therapeutic strategies in chondrosarcoma.

## MATERIALS AND METHODS

### Materials

ACDB (Figure 1A) was composed of benzofuran and other chemicals at the Graduate Institute of Pharmaceutical Chemistry, China Medical University (Taichung, Taiwan). α-minimum essential medium (MEM), Dulbecco's modified Eagle's medium (DMEM), fetal bovine serum (FBS) and other cell culture reagents were obtained from Gibco-BRL Life technologies (Grand Island, NY, USA). Rabbit polyclonal antibodies specific for poly adenosine diphosphate (ADP) ribose polymerase (PARP), GRP78, GRP94, calpain I, calpain II, Bid, Bcl-XL, Bak, Bax, Bcl-2, caspase-9, caspase-3, caspase-7, and anti- rabbit and anti-mouse IgG-conjugated peroxidase were obtained from Santa Cruz Biotechnology (Santa Cruz, CA). Mouse monoclonal antibodies specific for both cytochrome C and mitochondria control protein, voltage-dependent anion channels (VDAC), were obtained from Abcam (Cambridge, MA, USA). The ON-TARGET smart pool small interfering RNA (siRNA) against GRP78, GRP94, calpain I, calpain II, and control were obtained from Dharmacon (Lafayette, CO, USA). The Trizol, Lipofectamine 2000, and MMLV RT kit were purchased from Invitrogen (Carlsbad, CA, USA). 4',6-diamidino-2-phenylindole (DAPI), 3-[4, 5-dimethylthiazol-2-yl]-2, 5-diphenyltetrazolium bromide (MTT), 5,5',6,6'-tetrachloro-1,1',3,3′-tetraethyl benzimidazolylcarbocyanine iodide (JC-1), Fluo-3-pentaacetoxyMethyl ester (Fluo 3/AM), dihydroethidium (DHE), and dichlorodihydrofluorescein diacetate (H_2_DCFDA), 1,2-bis(2-aminophenoxy)ethane-N,N,N′,N′-tetraacetic acid tetrakis(acetoxymethyl ester) (BAPTA-AM), catalase, diphenyleneiodonium (DPI), catalase or N-acetylcysteine (NAC), and other chemicals were purchased from Sigma-Aldrich (St. Louis, MO, USA).

### Synthesized method of 2-amino-3-(2-chlorophenyl)-6-(4-dimethylaminophenyl)benzofuran-4-yl acetate (ACDB)

To the stirring solution of 5-[4-(dimethylamino)phenyl]cyclohexane-1,3-dione (0.346 g, 1.5 mmole) and 2-chloro-beta-nitrostyrene (0.187 g, 1 mmole) in tetrahydrofuran (50 mL) was added triethylamine (0.028 ml, 0.2 mmole) at room temperature for 9 h, and then added additional triethylamine (0.28 ml, 2 mmole), acetic anhydride (0.189 ml, 2 mmole) and dimethylaminopyridine (0.024 g, 0.2 mmole) for another 18 h. After the reacted mixture was concentrated in vaccum, the residue was subjected to column chromatography on silica gel with eluent of n-hexane:CH2Cl2 = 1:4 to afford the Rf = 0.23 fraction. The fraction was concentrated and recrystallization from acetonitril to obtain the pure purple-needle crystal ACDB (0.094 g, 23%). mp 201.1-201.7°C; ^1^H NMR (500 MHz, DMSO-*d*_6_) δ 7.53-7.55 (m, 1 H, ArH), 7.48 (d, *J* = 8.9 Hz, 2 H, 2'', 6''-H), 7.42 (d, *J* = 1.3 Hz, 1 H, 5-H), 7.34-7.40 (m, 2 H, ArH), 7.30-7.33 (m, 1 H, ArH), 6.99 (d, *J* = 1.3 Hz, 1 H, 7-H), 6.77 (d, *J* = 8.9 Hz, 2 H, 3′', 5′'-H), 6.34 (brs, 2 H, NH_2_), 2.92 (s, 6 H, 2×CH_3_), 1.54 (s, 3 H, CH_3_); ^13^C NMR (100 MHz, DMSO-*d*_6_) δ 168.9, 156.9, 151.2, 149.9, 141.0, 135.5, 133.8, 132.5, 131.5, 129.5, 129.0, 128.1, 127.2, 127.2, 127.2, 122.4, 114.4, 113.2, 113.2, 104.5, 86.2, 40.5, 40.5, 20.0; EIMS *m/z* 420.2 (M^+^). HPLC purity 99.40% (λ_max_ 324 nm).

### Cell culture

The human JJ012 cell line was kindly offered by Dr. Sean P Scully [53]. The human SW1353 cell line was purchased from the Bioresource Collection and Research Center in Taiwan. The cell was maintained in a humidified 37°C incubator with α- MEM/DMEM and 10% FBS containing 100 μg/ml streptomycin and 100 units/ml penicillin. Human primary chondrocytes were cultured as described previously [54]. Primary cultures of human chondrocytes were isolated from articular cartilage as previously described [41]. The cells were grown in plastic cell culture dishes in 95% air–5% CO_2_ with DMEM that was supplemented with 20 mM 4-(2-hydroxyethyl)-1-piperazine-ethanesulphonic acid (HEPES), 10% FBS, 2 mM-glutamine, penicillin (100 U/mL), and streptomycin (100 mg/mL).

### MTT assay

MTT assay was used to determine cell viability as described previously [41]. After treating with ACDB (3, 10, 30 μM) for 48 h, cells were incubated with MTT, and then analyzed the absorbance at 550 nm.

### DAPI staining

After treating with ACDB (3, 10, 30 μM) for 48 h, cells were fixed in a 70% ethanol solution, and then stained with a DNA-binding fluorescent dye, DAPI. Nuclear morphology was observed under fluorescence microscopy.

### Colony assay

For extended outcome of ACDB on chondrosarcoma, JJ012 cells (1,000/well) were stimulated with ACDB for 24 h. After formed colonies for 7 days, cells were marked with crystal violet and then dissolved in acetic acid to measure the absorbance at 550 nm.

### DNA fraction

In DNA laddering assay, the procedure was described previously [55]. Briefly, treated JJ012 cells were extracted the DNA with a mixture of isoamyl alcohol, chloroform, and phenol (1:24:25). The isolated DNA was analyzed electrophoretically on a 2% agarose gel and visualized under ultraviolet transillumination.

### Flow cytometric analysis

Apoptotic analysis was performed with flow cytometry through cell cycle and Annexin V-FITC kit as described previously [41]. JJ012 was treated with ACDB or vehicle in a dose-dependent concentrations and fixed in 70% chilled ethanol overnight. After washed with phosphate-citric acid buffer, cells were stained in the dark with propidium iodide (PI) buffer followed by fluorescence-activated cell sorting (FACS) and the Cellquest program (Becton Dickinson; Lincoln Park, NJ, USA). Double staining was measured according to Annexin V-FITC kit manufacturer (BD Biosciences, San Jose, CA).

### Mitochondrial membrane potential detection

Mitochondrial membrane potential activity was detected by the fluorescent probe, JC-1, as described previously [41]. JJ012 was stimulated with ACDB in a dose-dependent concentrations followed by incubation with JC-1 (10 mg/ml) for 30 min at 37°C in the dark. Cells were then re-suspended in specific solution and analyzed by FACS and the Cellquest program (Becton Dickinson, USA).

### Measurement of calcium concentration

Accumulating changes of calcium levels were measured as described previously [44]. Cells were plated with a density of 2x10^5^ cells/well and treated with ACDB for the indicated concentrations. Cells were collected and re-suspended in Fluo 3/AM (3 mg/ml) for 30 min at 37°C in the dark followed by analyzed with FACS and the Cellquest program (Becton Dickinson, USA).

### ROS assay with flow cytometry

Expression of intracellular O_2_^−^ and H_2_O_2_ were analyzed spectrofluorimetrically by oxidation of specific probes: DHE and H_2_DCFDA, which used 488 nm wavelengths. JJ012 was seeded 5x10^5^ cells/well in 6-well plates and treated with ACDB for specified time intervals. Cells were harvested, washed twice with chilled PBS, and stained with DHE (10 mM) or H_2_DCFDA (10mM) for 10 min in the dark. The fluorescent intensity of the samples were immediately measured by using the flow cytometry.

### Immunoblot analysis

The cellular lysates were collected as described previously [56]. Sample were separated into SDS–PAGE electrophoresis followed by transferred to Immobilon polyvinyldifluoride membranes (Millipore; Billerica, MA, USA). After protein blocking, the blots were incubated with specific primary antibodies followed by specific horseradish peroxidase-conjugated secondary antibodies. The blots were enhanced with chemiluminescence and visualized by using Fujifilm LAS-3000 chemiluminescence detection system (Fujifilm; Tokyo, Japan).

### Caspase activity assay

Caspase activity was determined according to caspase activity assay kit manufacturer (Abcam; Cambridge, MA, USA). Briefly, the cell lysates were collected and treated with peptide substrate, and then *p*-nitroaniline releasing from immunocomplexes was measured at 405 nm.

### *In vivo* mouse xenograft model

3-5 week male nude mice (BALB/c _nu/nu_) were obtained from the National Laboratory Animal Center (Taipei, Taiwan) and maintained in specific pathogen-free conditions. All protocols and guidelines were approved by Animal Care Committee of China Medical Taiwan University. JJ012 1x10^7^ cells in a volume of 160 μl were injected subcutaneously into the flanks of each mice. Once the tumors developed approximately 1000 mm^3^, mice were randomly divided into three groups (10 mice/group): the control group and the treatment groups, and treated with either different dose of ACDB or vehicle once daily for 20 days. The implanted tumor was measured three times a week. Tumor volume was calculated using the formula *V*=(*LW* ^2^)/2: where *V*, is the volume (mm^3^); *L*, the biggest diameter (mm); *W*, is the smallest diameter (mm).

ACDB-induced tumor apoptosis *in vivo* was detected by terminal deoxynucleotidyl transferase (TdT) deoxyuridine 5′-triphosphate (dUTP) nick-end labeling (TUNEL) assay as described previously [41]. TUNEL assay was measured according to the Apoptotic TUNEL assay kit (Trevigen, USA) manufacturer.

### Statistics

All quantified results are given as the means ± SEM by three experiments at least. Statistical comparison of two groups was performed the Student's t-test. Statistical comparisons of more than two groups were used one-way analysis of variance (ANOVA) with Bonferroni's post-hoc test. In all cases, p < 0.05 was defined statistically significant.

## SUPPLEMENTARY FIGURES


