# Supplementary material for: A novel benzofuran derivative, ACDB, induces apoptosis of human chondrosarcoma cells through mitochondrial dysfunction and endoplasmic reticulum stress
Source: Oncotarget. 2016 Nov 7;7(50):83530–43. doi: 10.18632/oncotarget.13171 (PMC5347786; doi:10.18632/oncotarget.13171)
Supplement: Supplementary file 1 [file oncotarget-07-83530-s001.pdf]

A novel benzofuran derivative, ACDB, induces apoptosis of human chondrosarcoma cells through mitochondrial dysfunction and endoplasmic reticulum stress

SUPPLEMENTARY FIGURES

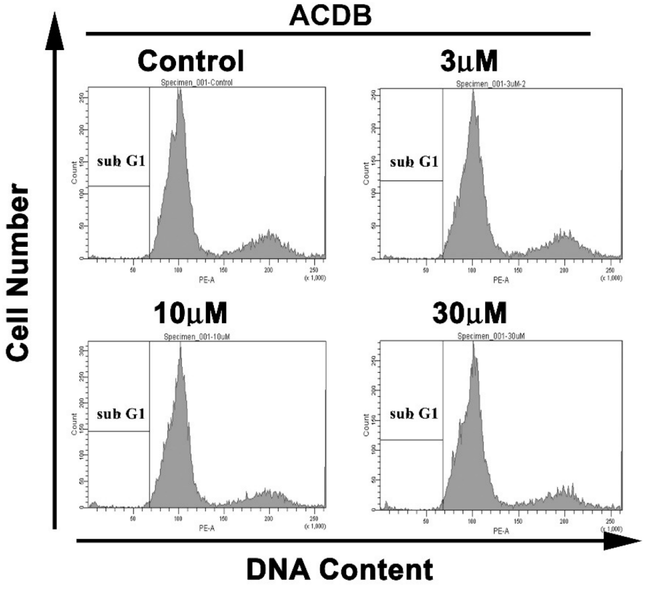

Supplementary Figure S1: Human primary normal chondrocytes were incubated with ACDB (10 µM) for 24 hours. The cell cycle analysis (PI staining) was examined by flow cytometry.

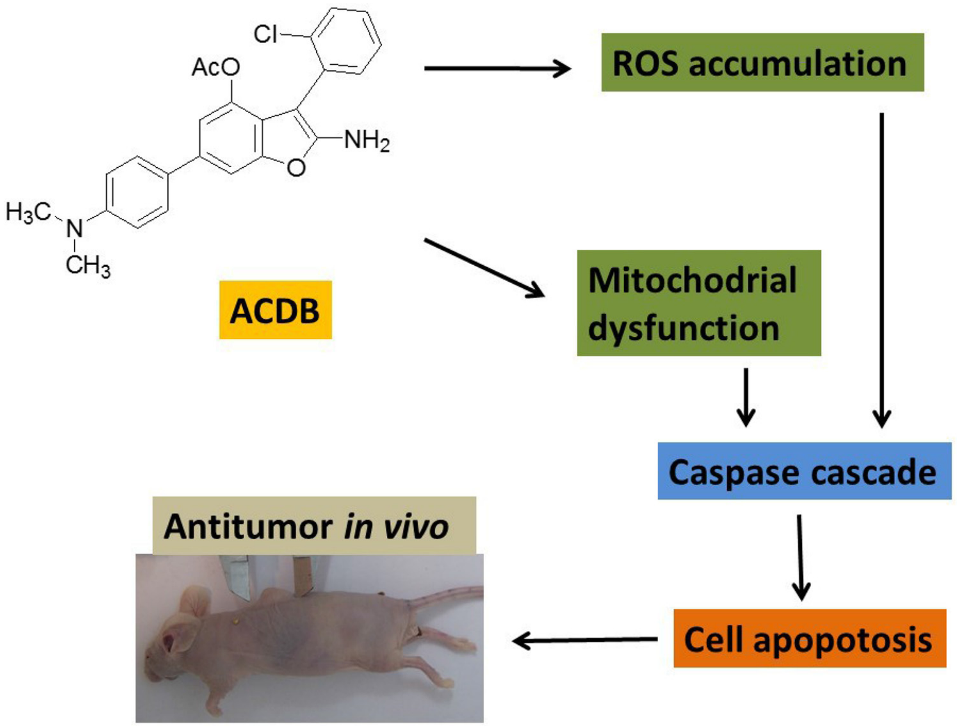

Supplementary Figure S2: Diagram presentation of the signaling pathways involved in ACDB-triggered apoptosis of human chondrosarcoma cells *in vitro* and *in vivo*.
